# ROS and RNS: key signalling molecules in plants

**DOI:** 10.1093/jxb/ery198

**Published:** 2018-06-19

**Authors:** Ismail Turkan

**Affiliations:** Ege University, Faculty of Science, Department of Biology, Izmir, BO, Turkey

**Keywords:** Abiotic stress, biotic stress, defence, development, free radicals, oxygen toxicity, reactive nitrogen species (RNS), reactive oxygen species, reactive oxygen species (ROS)

**Both reactive oxygen species (ROS) and reactive nitrogen species (RNS) are important signals in plants and key regulators of a variety of processes including metabolism, growth and development, response to abiotic and biotic stresses, solute transport, autophagy and programmed cell death (PCD). The reviews and original research in this special issue reflect a burgeoning area of investigation, and highlight the latest thinking on new roles and interactions of ROS and RNS, the basis of their specificity and flexibility, and emergent fields.**

Since Gerschman *et al.* (1954) advanced the idea of oxygen radicals as harmful molecules and the discovery of the enzyme superoxide dismutase (McCord and Fridovich, 1969), with the superoxide anion radical (O_2_^.–^) as a substrate, research on reactive oxygen species (ROS) has grown exponentially. Next key findings were the identification of hydrogen peroxide (H_2_O_2_) as a by-product of aerobic metabolism (Sies and Chance, 1970) and demonstration of O_2_^.–^ production in illuminated chloroplasts (Asada *et al.*, 1974), and then later with the establishment of the ‘oxidative stress’ concept (Sies, 1985) researchers focused on the elucidation of mechanisms that allow the plant to cope with the accumulation of ROS. These efforts led to the identification of various enzymatic and non-enzymatic antioxidants (Mittler *et al.*, 2004). However, identification of plant homologues of NADPH oxidases (Sagi and Fluhr, 2001) and the plant thioredoxin/peroxiredoxin system as redox regulators of different metabolic processes, mainly via thiol switches, established a signalling role for ROS and the cellular redox state (Dietz, 2003; Dietz, 2014). Accumulated evidence indicated that ROS not only damage molecules but are required at low levels and can act as signals, giving rise to the ‘redox signalling’ concept (Foyer and Noctor, 2003).

RNS in plants had already been identified as early as the 1960s (Fewson and Nicholas, 1960), but they did not receive as much attention as their oxygen counterparts (ROS) until the end of the 1990s (Leshem and Haramaty, 1996; Noritake *et al.*, 1996; Delledonne *et al.*, 1998; Durner *et al.*, 1998). These pioneering works established that nitric oxide (NO) acts as a signalling molecule during plant pathogenesis and again attracted the attention of researchers looking to elucidate the physiological roles of RNS in plants. However, in contrast to ROS, which were first recognized as damaging molecules and then signals, RNS were first recognized through their signalling role and the term ‘nitrosative stress’ did not emerge in the plant biology literature until the 2000s. It is now well known that ROS and RNS are important signals in plants and key regulators of a variety of processes including metabolism, growth and development, response to abiotic and biotic stresses, solute transport, autophagy and PCD (Foyer and Noctor, 2015; del Rio, 2015; see also Turkan, 2017). Still, after more than 50 years of intense research, new roles at both physiological and molecular levels are being postulated for ROS and RNS, emphasizing their central role in the functioning of plant cells.

## Specificity in ROS signalling: are we there yet?

ROS signals are complex in a variety of ways. First, different types of ROS such as O_2_^.–^, ^1^O_2_, HO^.^ or H_2_O_2_ have different half-lives and affinities for biological molecules. For example ^1^O_2_ and HO^.^ are very short-lived and highly reactive, but H_2_O_2_ has lower reactivity and a longer half-life; this makes H_2_O_2_ a more suitable signalling molecule, while the former examples are known to signal with their breakdown products. Second, their production dynamics and subcellular localization can differ according to the physiological state of the plant cell. Both ^1^O_2_ and O_2_^.–^ can be produced in chloroplasts when the balance between light reactions and Calvin reactions is impaired due to excess excitation of the photosystems or insufficient supply of CO_2_; O_2_^.–^ can be produced in mitochondria when the electron transport chain is overloaded; H_2_O_2_ can be produced in all the compartments with the dismutation of O_2_^.–^. Furthermore, photorespiratory H_2_O_2_ produced in peroxisomes, especially in C_3_ plants, is an important source of ROS. In addition to the particular type of molecule and spatial distribution of ROS, signalling is furthermore complicated by variation in time, i.e. the duration of exposure to ROS or ROS-inducing environmental stimuli.

Although general mechanisms and dynamics of ROS production have been elucidated in plant cells under different environmental stimuli and at different developmental stages, we are far from a full picture. Reviews by Turkan *et al.* (2018), Ozgur *et al.* (2018) and Krasensky-Wrzaczek and Kangasjärvi (2018) illustrate this well. Turkan *et al.* (2018) provide a comparative overview of redox regulation in C_3_ and C_4_ plants, with particular emphasis on the mesophyll and bundle sheath cells of the C_4_ plants, a topic which is gaining a wide audience due to efforts to convert C_3_ plants to C_4_ plants to increase yield (Furbank, 2016). The review focuses on linear and cyclic electron transport in the chloroplasts of C_3_ and C_4_ plants (also mesophyll and bundle sheath cells) and discusses implications for photosynthetic light reactions, ROS production dynamics, antioxidant defence, and thiol-based redox regulation. In this sense, it draws attention to the issue that it appears impossible to utilize efficient C_4_ photosynthesis without understanding its exact redox needs, which will certainly be a topic of interest in the future.

Similarly, Ozgur *et al.* (2018) review a previously unexplored topic, the connections between endoplasmic reticulum (ER) stress (accumulation of unfolded proteins in the ER lumen), the unfolded protein response, and ROS. The focus is on mechanisms of ROS production originating from the ER, the interaction between ER stress and overall ROS signalling processes in the cell, and the interaction of ER stress with other organellar ROS signalling pathways such as those in the mitochondria and chloroplasts.

In contrast, Krasensky-Wrzaczek and Kangasjärvi (2018) focus on the temporal dynamics of ROS production rather than their subcellular localization. They provide an overview of ROS production, redox regulation and antioxidant defence in plants grown under short and long days. Further, they relate the ROS production with the circadian clock, based on both transcription-translation feedback loops and the non- transcriptional oscillating redox-based clock (oxidation status of peroxiredoxins). The authors also provide a new concept as to how PCD is regulated in response to different day lengths via ROS, glutathione (GSH) and salicylic acid, which seems to be of paramount importance during pathogen defence.

Specificity in ROS signalling depends on the type, site, amount and duration of the ROS signature, as well as the ability to regulate gene expression in response to the perceived stimuli. Huaming *et al.* (2018) provide an overview of current knowledge on the control of ROS and thiol-dependent transcriptional machinery. Detailed information is provided on oxidative stress-responsive *cis*-regulatory elements, ROS-sensitive transcription factors and ROS-responsive transcripts. In addition, using *cat2* mutants as a model system, they assess the impact of redox perturbations and oxidative stress on transcriptome adjustments and discuss how redox homeostasis can modify the various parts of the transcriptional machinery.

As second messengers, besides induction of specific signals, ROS can act during the acquisition of cross-stress tolerance, which usually occurs during intense and prolonged exposure to oxidative stress. Locato *et al.* (2018) discuss the retrograde signalling mechanisms, especially from chloroplasts and mitochondria, that are involved in acquiring cross-tolerance. In addition, they provide an overview of new developments in research on imprinting stress memory, including the interaction between epigenetic mechanisms and redox metabolism during stress responses, which involve DNA methylation and histone modifications, as well as the emerging roles of GSH in histone glutathionylation and regulation of histone-modifying enzymes.

A final review related to ROS and oxidative stress, by Kim *et al.* (2018), relates to the recently isolated *Orange* gene (encoding a holdase chaperone protein) that is responsible for regulation of carotenoid homeostasis. Since carotenoids are indispensable for organisms that use oxygenic photosynthesis and are vital components for protection of photosynthetic machinery from oxidative damage, the authors discuss the rationale of increasing plant tolerance by controlling carotenoid biosynthesis via *Orange*.

Research articles in this special issue cover various areas of plant biology reflecting again the involvement of ROS in a plethora of cellular functions, including regulation of Na^+^ transport by ROS (Niu *et al.*, 2018) or redox-active ascorbate (Makavitskaya *et al.*, 2018; see also the Insight article by Pottosin and Zepeda-Jazo, 2018) and changes in the structure and function of a protein malate dehydrogenase with oxidative modifications (Huang *et al.*, 2018).

## Progress with RNS

Unlike for ROS, production mechanisms of RNS in plants remain in part unresolved and this is an active area of research. Also, in the past decade, research on the signalling mechanisms mediated by RNS have accelerated, partly due to the elucidation of their interactions with ROS. NO is a small signalling molecule involved in many physiological aspects of plant growth, as comprehensively outlined by Astier *et al.* (2018). These authors also highlight what we know about NO production and signalling in plants, as well as looking at the gaps in our knowledge. Complementing this coverage, Gupta *et al.* (2018) describe the regulation of NO production by the mitochondrial electron transport chain, especially complex I, alternative NAD(P)H dehydrogenases, complex II, alternative oxidase, complex III, cytochrome c and complex IV. These authors also highlight the importance of the relationship between RNS and respiration processes in responses to environmental stresses, as well as our lack of knowledge on the regulatory role of NO in mitochondrial metabolism during stress.

The regulation of cellular metabolism and signalling via post-translational modifications such as *S*-nitrosylation is one of the most interesting roles of RNS. *S*-nitrosylation of non-enzymatic antioxidant glutathione activates the production of S-Nitrosoglutathione (GSNO), which is also an important molecule involved in responses to abiotic stress and plant immunity (Begara-Morales *et al.*, 2018). GSNO turnover mechanisms in plants and various methods used in the detection of NO and *S*-nitrosothiol (SNO) levels are addressed by Begara-Morales *et al.* (2018). The authors also provide a list of SNO and GSNO levels in various plant tissues and species measured using different methods. Umbreen *et al.* (2018) review the specificity of NO signalling and especially elaborate on the role of protein denitrosylation and its interaction with RNS and ROS. Furthermore, these authors highlight the removal of *S*-nitrosylation via GSNO reductase and thioredoxin h5 (Trxh5) as being as important as generation of *S*-nitrosylation during RNS signalling. The final review on RNS in the special issue, by Corpas *et al.* (2018), provides an overview of the involvement of H_2_O_2_, NADPH, NO, peroxynitrite (ONOO^–^) and SNOs during fruit ripening. They follow a translational approach and discuss the use of basic research on ROS/RNS interaction to improve fruit yield and quality of pepper and tomato.

## Perspectives

As can be seen, new roles and interactions are being postulated for ROS and RNS, increasing our understanding of the underlying mechanisms allowing signalling, yet we still lack the fundamental knowledge as to how these reactive molecules act as such specific signals. To dissect both their specificity and their flexibility, recently attention has been given to their interactions at the molecular level, following technological advances. Another emerging area of research is the dissection and study of the roles of breakdown products as a result of biomolecule and ROS/RNS interaction. To conclude, it is of paramount importance to highlight the importance of physical interactions between different organelles to understand the specificity of the ROS/RNS signals, since the site of production and target for regulation must be in close proximity due to the reactive nature of these molecules.
